# 

**Published:** 1909-11

**Authors:** 


### BOOKS RECEIVED.

The Practical Medicine Series. Comprising ten volumes on the year's progress in medicine and surgery, under the general charge of Gustavus P. Head, M.D., Professor of Laryngology and Rhinology at the Chicago Postgraduate Medical School. Vol. VII. Pediatrics. Edited by Isaac A. Abt. Orthopedic surgery. Edited by John Ridlou. 1909. Chicago: The Year Book Publishers. (Price, $1.25.)

The Practice of Medicine. By James Tyson, M.D., Professor of Medicine in the University of Pennsylvania and Physician to its Hospital. Fifth edition. Octavo, pp. 1438. Illustrated. Philadelphia: P. Blakiston's Son & Co. 1909. (Cloth, $5.50.)

A Handbook of Medical Diagnosis. By J. C. Wilson, M.D., Professor of the Practice of Medicine and Clinical Medicine in Jefferson Medical College and Physician to its Hospital. Octavo, pp. 1435. Illustrated. Philadelphia and London: J. B. Lippincott Co. 1909.

A Practical Treatise on Diseases of the Skin. For the use of students and practitioners. By J. Nevins Hyde, A.M., M.D., Professor of Dermatology and Venereal Diseases in the University of Chicago, Medical Department (Rush Medical College). Eighth edition, thoroughly revised and much enlarged. Octavo of 1137 pages, with 223 engravings and 58 full-page plates, in colors and monochrome. Lea & Febiger, Philadelphia and New York, 1909. (Cloth, $5.00, leather, $6.00, net prices.)

A Textbook on the Principles and Practice of Surgery. By George Emerson Brewer, M.D., Professor of Clinical Surgery in the College of Physicians and Surgeons, New York. Octavo, 908 pages, 415 engravings and 14 full-page plates. Lea & Febiger, Philadelphia and New York, 1909. (Cloth, $5.00; leather, $6.00, net prices).

A Manual of Otology. By Gorham Bacon, A.M., M.D., Professor of Otology in the College of Physicians and Surgeons, Columbia University, New York. With an Introductory Chapter by Clarence J. Blake, M.D., Professor of Otology in the Harvard Medical School, Boston. Fifth edition, thoroughly revised. 12mo, 500 pages, 147 engravings and 12 plates. Lea & Febiger, Philadelphia and New York, 1909. (Cloth, $2.25, net.)

Organic and Functional Nervous Diseases. A Textbook of Neurology. By Allen Starr, M.D., Ph.D., LL.D., Sc.D., Professor of Neurology, College of Physicians and Surgeons, New York. Third edition. Octavo, 904 pages, with 300 engravings and 29 plates in colors or monochrome. Lea & Febiger, Philadelphia and New York, 1909. (Cloth, $6.00; leather, $7,00, net prices.)

Practical Points in the use of x-ray and High-Frequency Currents by Aspinwall Judd, M.D., formerly Radiologist Post-Graduate Medical School and Hospital, New York. New York: Rebman Co. 1909. (Cloth, $1.50.)

Renal, Ureteral, Perineal and Adrenal Tumors and Actinomycosis and Echinococcus of the kidney. By Edgar Garceau, M.D., Visiting Gynecologist to St. Elizabeth's Hospital, and to the Boston Dispensary, Boston. Octave, pp. 434. Illustrated. New York and London: D. Appleton & Co. 1909. (Cloth, $5.00.)

Chemical and Microscopical Diagnosis. By Francis Carter Wood, M.D., Professor of Clinical Pathology, College of Physicians and Surgeons, New York. Octavo, pp. 771. Illustrated. New York and London: D. Appleton & Co. 1909. (Cloth, $5.00.)

Diagnostics of Internal Medicine. By Glentworth R. Butler, M.D., Physician-in-Chief to the Methodist Episcopal Hospital, Brooklyn. Third edition. Octavo, pp. 1227. Illustrated. New York and London: D. Appleton & Co. 1909. (Cloth, $6.00.)

